# First-Trimester Preeclampsia Screening During the COVID-19 Pandemic: A Quality Improvement Comparison of the National Institute for Health and Care Excellence (NICE) Guidelines vs. Simplified Fetal Medicine Foundation Algorithm

**DOI:** 10.7759/cureus.81371

**Published:** 2025-03-28

**Authors:** Konstantinos Malligiannis Ntalianis, Christina Resta, Lama Daher, Sundararajah Raajkumar, Maria Saridi, Pavlos Sarafis, Theocharis I Konstantinidis

**Affiliations:** 1 Obstetrics and Gynaecology, Mid & South Essex NHS Foundation Trust, Southend-On-Sea, GBR; 2 Obstetrics and Gynaecology, Guy's and St Thomas' NHS Foundation Trust, London, GBR; 3 Obstetrics and Gynaecology, Mid & South Essex NHS Foundation Trust, Southend-on-Sea, GBR; 4 Department of Social Sciences, Hellenic Open University, Patras, GRC; 5 Department of Nursing, University of Thessaly, Lamia, CYP; 6 Department of Nursing, Hellenic Mediterranean University, Heraklion, GRC

**Keywords:** prediction, preeclampsia, pregnancy, prevention, screening

## Abstract

Objective: To evaluate the effectiveness of the National Institute for Health and Care Excellence (NICE) guidelines versus the mini-combined test for preeclampsia screening at 11-14 weeks of gestation, considering COVID-19 restrictions.

Methods: This study included women ≥18 years old with singleton pregnancies attending routine antenatal visits between May 1 and September 1, 2021. Data collected included maternal characteristics, medical history, uterine artery pulsatility index (PI), and pregnancy-associated plasma protein-A (PAPP-A) levels. Both NICE and Fetal Medicine Foundation (FMF) methods were used to classify high and low-risk cases.

Results: The mini-combined method showed 50% sensitivity and 89.9% specificity at a 1:100 cutoff for all preeclampsia cases. An optimal cutoff of 1:165.5 yielded 70.5% sensitivity and 80.9% specificity. NICE’s method demonstrated 22.7% sensitivity and 90.9% specificity.

Conclusion: The mini-combined screening method using the FMF algorithm outperforms the NICE method in preeclampsia screening. Implementing the full FMF method, including mean arterial pressure (MAP) and placental growth factor (PLGF), is recommended based on superior performance and international literature support.

## Introduction

Eclampsia, first described by Hippocrates in ancient Greece, refers to the sudden onset of seizures in pregnant women [1,2].

Hypertensive disorders affect 10% of pregnancies, defined by The International Society for the Study of Hypertension in Pregnancy (ISSHP) as blood pressure ≥140/90 mmHg after 20 weeks gestation. This includes gestational hypertension and preeclampsia. Chronic hypertension can be identified either prior to 20 weeks of gestation or prior to conception. Preeclampsia is specifically marked by the emergence of high blood pressure after 20 weeks, along with signs of maternal organ dysfunction and/or uteroplacental dysfunction, or proteinuria (≥300 mg albumin/24h or protein-creatinine ratio ≥30 mg/mmol). Systemic organ failure may include acute kidney injury, neurological disorders, liver injury, blood disorders, and placental insufficiency. The etiology is unclear but may involve impaired placentation, oxidative stress, and imbalanced trophoblast-derived factors like soluble fms-like tyrosine kinase-1 (sFLT1) and placental growth factor (PLGF).

Preeclampsia poses significant risks to both mothers and their children. Mothers face a two- to four-fold increased risk of long-term hypertension, doubled cardiovascular mortality, and a 1.5-fold higher stroke risk. Fetuses may experience intrauterine growth restriction, preterm birth, oligohydramnios, placental abruption, fetal distress, and in-utero death. Additionally, children exposed in utero may develop early onset hypertension and increased ischemic heart disease and stroke risk [3-5].

Risk factors include a history of preeclampsia, autoimmune disease, chronic kidney disease, diabetes, chronic hypertension, advanced maternal age, high BMI, multiple pregnancies, and specific ethnic backgrounds. Patients require regular monitoring. Mild hypertension (≥140/90 mmHg) is managed with antihypertensives like labetalol or nifedipine, aiming for blood pressure ≤135/85 mmHg. Severe hypertension (systolic ≥160 mmHg or diastolic ≥110 mmHg) requires urgent treatment and hospitalization.

Delivery timing should be guided by HYPITAT-I and PHOENIX trials, which suggest that delivery before 37 weeks of gestation carries less risks for maternal mortality and morbidity for pregnancies affected by preeclampsia as well as that there is no negative impact on the neurodevelopment of those newborns as investigated by the age of 2. Postpartum blood pressure peaks between days 3 and 6, necessitating close monitoring and continuation of antihypertensives if needed. Long-term, women with a history of preeclampsia should receive cardiovascular follow-up postpartum [6].

Hypertensive disorders of pregnancy should be recognized as predisposing factors of long-term maternal cardiovascular morbidity and therefore follow-up should be implemented 6-12 weeks postpartum and then periodically, preferably annually, after a pregnancy has been complicated by hypertensive disorders [7,8].

Preeclampsia significantly inflates healthcare expenses. In Europe, preeclampsia-complicated pregnancies cost about twice as much as uncomplicated ones (€5,243 vs €2,452). U.S. figures show an even starker contrast: $41,000 for preeclampsia cases versus $13,000 for normal pregnancies. A King’s College London review confirms this 1:2 or 1:3 cost ratio. Low-risk pregnancies cost between £1,381 and £4,145, while high-risk cases can reach £16,205 [9-11].

It becomes apparent that the detection of the high-risk group of pregnant women to develop preeclampsia as well as the prevention of the onset, especially the early/preterm preeclampsia is crucial. The ASPRE study demonstrated that aspirin reduces preterm preeclampsia incidence by 62% when started early in high-risk women. Adherence to aspirin therapy plays a crucial role, with efficacy increasing to 75% in those with ≥90% adherence [12].

The National Institute for Health and Care Excellence (NICE) advises identifying high-risk women for preeclampsia based on medical history and characteristics, detecting about 40% of preterm and 35% of term cases. High-risk women, such as those with chronic hypertension or autoimmune diseases, should take 75-150 mg of aspirin daily from 12 to 36 weeks of gestation. Intermediate-risk women are also advised to take aspirin if they have at least two risk factors. These factors include a first pregnancy, age over 40, a long interval since the last pregnancy, BMI of 35 kg/m^2^ or more at the first visit, family history of preeclampsia, or multiple pregnancies [13-14].

The SPREE trial suggests an alternative screening method, a combined method involving maternal characteristics, mean arterial pressure (MAP), uterine artery pulsatility index (UtA-PI), and PLGF measurement at 11-14 weeks. This approach predicts 90% of early onset cases and 75% of preterm preeclampsia cases. It also applies to twin pregnancies, considering revised UtA-PI, MAP, and PLGF values, by using the new value distributions log10 multiples of the median (MoM) of UtA-PI, MAP, and PLGF according to gestational age at delivery with preeclampsia, to calculate the risk [15]. Whether aspirin prevents preeclampsia in twin pregnancies as it does in singletons remains to be proven in an ongoing international randomized trial.

Trials based on the findings of the SPREE study showed that a mini-combined method using maternal history, MAP, UtA-PI, and PAPP-A instead of PLGF, typically recorded during routine first-trimester screening, showed comparable efficacy to the full model in predicting preeclampsia. This simplified approach may be more feasible for healthcare settings [13,16].

## Materials and methods

We conducted this retrospective study, part of our department’s Quality Improvement Project, comparing the NICE guidelines and the mini-combined method for first-trimester preeclampsia prediction. The sample included women attending routine antenatal check-ups at Mid & South Essex NHS Foundation Trust between May 4, 2021, and September 1, 2021, with data collected until April 27, 2022.

Inclusion criteria mandated that participants be at least 18 years old with singleton pregnancies featuring a viable, non-anomalous fetus detected on an 11-14-week ultrasound. Women who were unconscious, critically ill, or suffering from severe mental disorders were excluded from the study.

Recorded parameters included a comprehensive range of maternal characteristics such as date of birth/age, height, weight, race, family history of preeclampsia, smoking history, method of conception, and obstetric history. In addition, medical history was thoroughly documented, covering chronic hypertension, type I or II diabetes, autoimmune diseases, and chronic kidney disease. Ultrasound assessments focused on the UtA-PI, while biochemical evaluations involved the measurement of pregnancy-associated plasma protein-A (PAPP-A), expressed in MoM.

Each participant was assessed for preeclampsia risk using both the NICE guidelines and the Fetal Medicine Foundation’s (FMF) algorithm of the combined method [2].

Due to COVID-19 restrictions, MAP measurement was not feasible as the first antenatal booking appointment was conducted via telephone consultation. Unfortunately, this limitation may have impacted the overall performance of both screening methods during this study. However, our main goal was to investigate if the combined method, even with this limitation, would be superior to the NICE screening method.

Therefore, regarding the calculation of the risk of developing preeclampsia for the combined method, the online FMF algorithm was used taking into consideration: Pregnancy type and dating, maternal characteristics, obstetric and medical history, mean uterine artery PI, and PAPP-A.

## Results

A study of 646 women, averaging 30.4 years old, examined sociodemographic data, BMI, and lifestyle habits. Predominantly white (93.2%), the group had a mean BMI of 27.6 kg/m², with 31.3% being overweight. Most participants (91.8%) were non-smokers, and none consumed alcohol. Individual medical histories were also collected. Table 1 provides insight into patients’ characteristics.

**Table 1:** Maternal characteristics

Parameters		N	%
Maternal age, mean (SD)		30.4 (5.1)	
Race	White/Caucasian	602	93.2
	Black/African	13	2.0
	East Asian	10	1.5
	South Asian	16	2.5
	Mixed	5	0.8
Conception Method			
	Spontaneous	626	96.9
	IVF	18	2.8
	Other methods of assisted reproduction	2	0.3
BMI (kg/m^2^), mean (SD)		27.6 (6.2)	
BMI levels	Underweight (<18.5)	14	2.2
	Normal (18.5 to 24.9)	243	37.7
	Overweight (25 to 29.9)	202	31.3
	Obese (≥ 30)	186	28.8
Smoking	No	593	91.8
	Yes	44	6.8
	Stopped	9	1.4
Alcohol	No	646	100.0
	Yes	0	0.0
Obstetric History			
Parity	Nulliparous	297	46
	Multiparous	349	54
PE in a previous pregnancy		5	0.8
Family history of PE		2	0.3
Medical History			
Diabetes	No	642	99.4
	Yes	4	0.6
Chronic hypertension	No	646	100.0
	Yes	0	0.0
Nephropathy	No	646	100.0
	Yes	0	0.0
Antiphospholipid syndrome (APS)/Systemic lupus erythematosus (SLE)	No	646	100.0
	Yes	0	0.0
Other: Penicillin allergy, burning in the cervix, depression, intracranial hypertension, large loop excision of the transformation zone (LLETZ), polycystic ovaries, rheumatoid arthritis, supraventricular tachycardia, ulcerative colitis, vaginal prolapse, hemophilia		23	3.6

Sixty-two patients were affected by hypertension in pregnancy either gestational hypertension or preeclampsia. Preeclampsia during pregnancy was observed in 44 participants and gestational hypertension was experienced by 18 participants, which constitutes 6.8% and 2.8% of the sample, respectively.

Details of the participants who developed preeclampsia are given in Table 2. The mean gestational age at onset of preeclampsia was 37.3 weeks (SD =4.2 weeks). Pulmonary edema was experienced by 4.5%, and brain and/or vision symptoms were experienced by 14% of participants with preeclampsia, eclampsia, renal failure, or HELLP syndrome was experienced by 2.3% respectively. Antihypertensive treatment for hypertension was received by 34/44 women (i.e., 77.3%).

**Table 2:** Data on participants who developed preeclampsia

Parameter	N (%)	Mean (SD)	Median (IQR)
Gestational age in weeks at onset of preeclampsia	—	37.3 (4.2)	—
SBP (mmHg)	—	145.2 (5.8)	—
DBP (mmHg)	—	89.9 (6.9)	—
Platelets	—	217.4 (63.8)	—
Creatinine (µmol/L)	—	62.7 (15.8)	—
UPCR	—	114 (222.5)	48 (32–100)
AST (U/L)	—	31.8 (50.6)	21 (16.5–28)
ALT (U/L)	—	29.5 (56.5)	15 (11–24)
Pulmonary edema	2 (4.5%)	—	—
Brain or vision symptoms	6 (14%)	—	—
Eclampsia	1 (2.3%)	—	—
HELLP syndrome	1 (2.3%)	—	—
Renal failure	1 (2.3%)	—	—
Antihypertensive treatment – None	10 (22.7%)	—	—
Antihypertensive treatment – Labetalol	19 (43%)	—	—
Antihypertensive treatment – Nifedipine	10 (22.7%)	—	—
Antihypertensive treatment – Labetalol + Nifedipine	4 (9%)	—	—
Antihypertensive treatment – Enalapril (postpartum)	1 (2.3%)	—	—

The 39 pregnant women with preeclampsia were white (88.6%), 3 Black/African (6.8%), 1 East Asian (2.3%) and 1 South Asian (2.3%), while a total of 34 of the 44 women with preeclampsia had a BMI > 25 (77.3%).

Regarding the obstetric outcome and the newborns, information is given in Table 3. The mean birth weight was 3388.7 g ( SD =535.3 gr ). A low-birth-weight infant was born in 60 cases, 9.3%. Of the low-birth-weight infants, 12 were in pregnancies that were affected by preeclampsia. 3.6% of newborns were admitted to neonatal ICU (NICU). Sadly, two intrauterine deaths were recorded.

**Table 3:** Information about obstetric outcomes and newborns

Parameter	N (%)	Mean (SD)
Gestation week at delivery < 37	31 (4.8%)	—
Gestation week at delivery ≥ 37	615 (95.2%)	—
Birth - Vaginal	424 (65.6%)	—
Birth - Emergency cesarean section	100 (15.5%)	—
Birth - Planned cesarean	122 (18.9%)	—
Livebirth	644 (99.7%)	—
Intrauterine/Neonatal death	2 (0.3%)	—
Admission to Neonatal unit/Special care	23 (3.6%)	—
Low-birth-weight newborn (SGA) - No	584 (90.4%)	—
Low-birth-weight newborn (SGA) - Yes	60 (9.3%)	—
Low-birth-weight newborn with PE	12 (20%)	—
Gender of newborn - Female	325 (50.3%)	—
Gender of newborn - Male	321 (49.7%)	—
Apgar 1'	—	8.8 (1.1)
Apgar 5'	—	9.8 (0.8)
Arterial pH	—	7.2 (0.5)
Venous pH	—	7.2 (0.7)
Birth weight (g)	—	3388.7 (535.3)

Receiver-operating characteristic (ROC) analysis for preeclampsia

Using receiver-operating characteristic (ROC) analysis, the predictive value of the NICE risk and FMF risk indices were calculated, described in Table 4.

**Table 4:** Comparison of the prognostic value of the two methods for predicting preeclampsia overall, <37 and >37 weeks FMF: Fetal Medicine Foundation; PPV: positive predictive value; NPV: negative predictive value; AUC: area under the curve

	AUC*	95% CI	P	Cutoff	Sensitivity (%)	Specialty (%)	PPV^#^ (%)	NPV^$^ (%)	Accuracy (%)
Sample total									
NICE risk	0.57	0.47 – 0.66	0.132	-	22.7	90.9	15.4	94.1	86.2
FMF risk	0.76	0.66 - 0.84 _	<0.001	< 165.5	70.5	80.9	21.2	97.4	80.2
FMF risk	0.70	0.61-0.79	<0.001	100	50	89.9	0.27	0.96	0.87
<37 weeks									
NICE risk	0.59	0.26 – 0.92	0.576	-	25.0	92.6	33.3	89.3	83.9
FMF risk	0.88	0.68 – 1.00	0.014	< 74	75	100	-^£^	87.1	87.1
≥37 weeks									
NICE risk	0.57	0.47 – 0.67	0.160	-	22.5	90.8	14.5	94.4	86.3
FMF risk	0.74	0.64 -0.84	<0.001	< 165.5	70	80.9	20.3	97.5	80.2
*Area under the curve ^#^Positive predictive value ^$^Negative predictive value ^£^could not be calculated due to null values									

**Figure 1:**
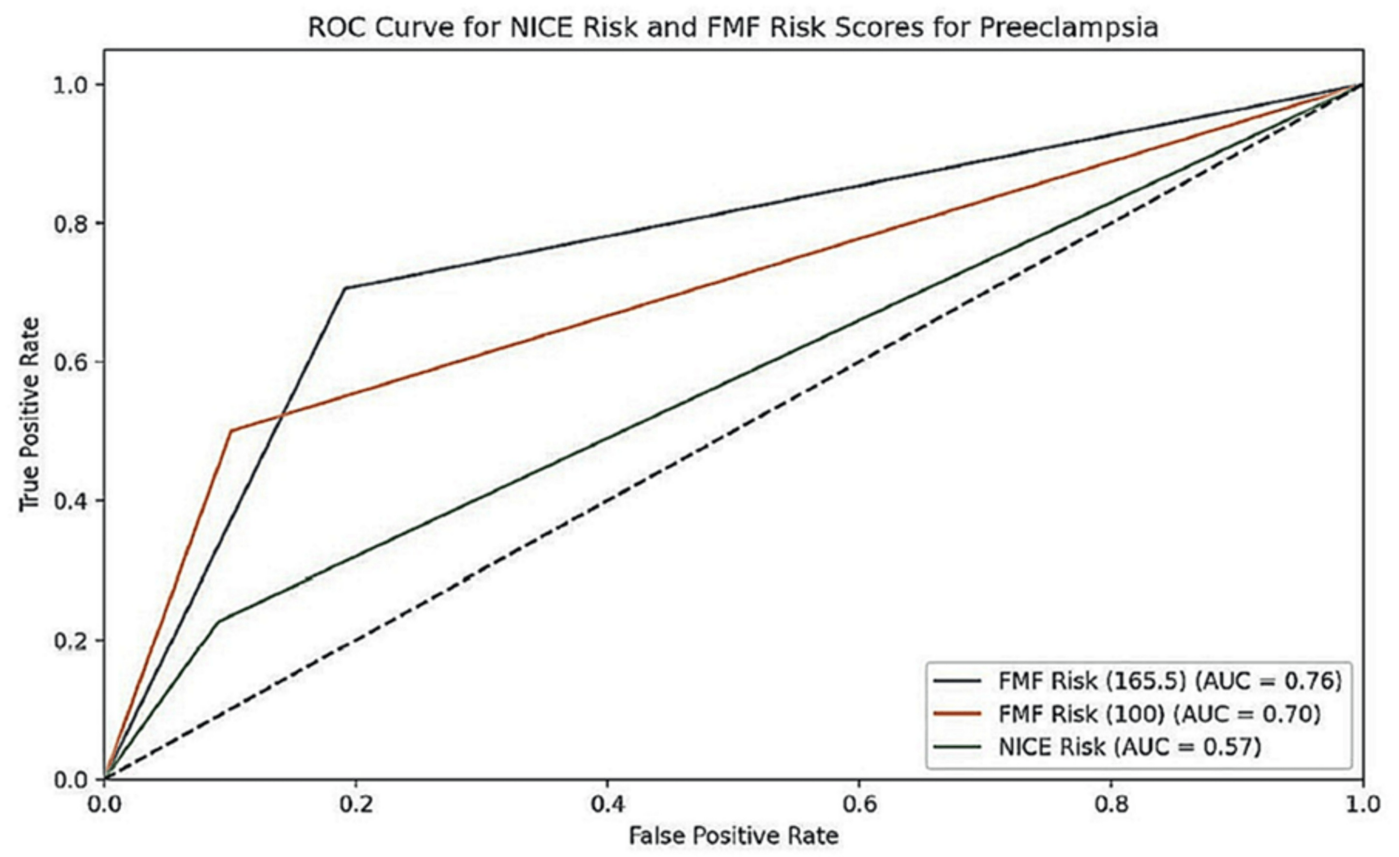
ROC curve for the predictive ability of NICE and risk indices for preeclampsia overall ROC: receiver-operating characteristic; NICE: National Institute for Health and Care Excellence

**Figure 2:**
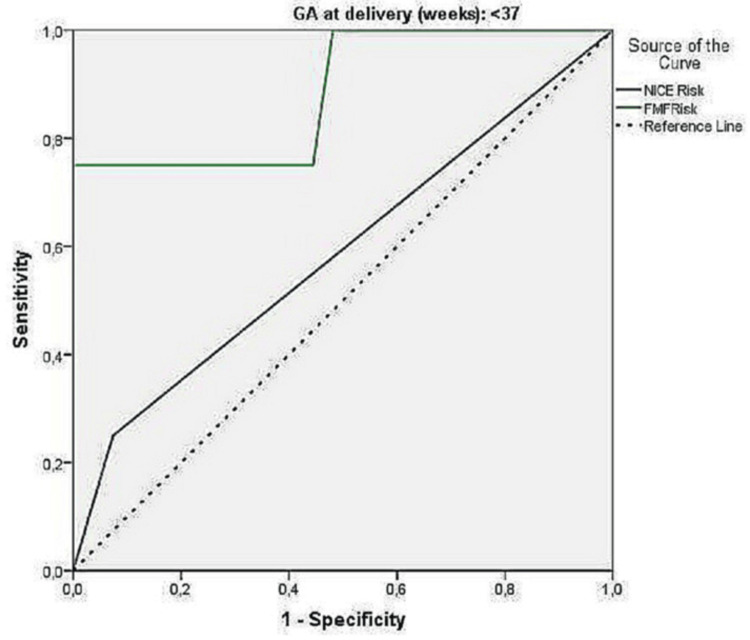
ROC curve for the predictive ability of NICE risk and FMF risk indices for preeclampsia ≤ 37 weeks (FMF cutoff 165.5) ROC: receiver-operating characteristic; NICE: National Institute for Health and Care Excellence; FMF: Fetal Medicine Foundation

**Figure 3:**
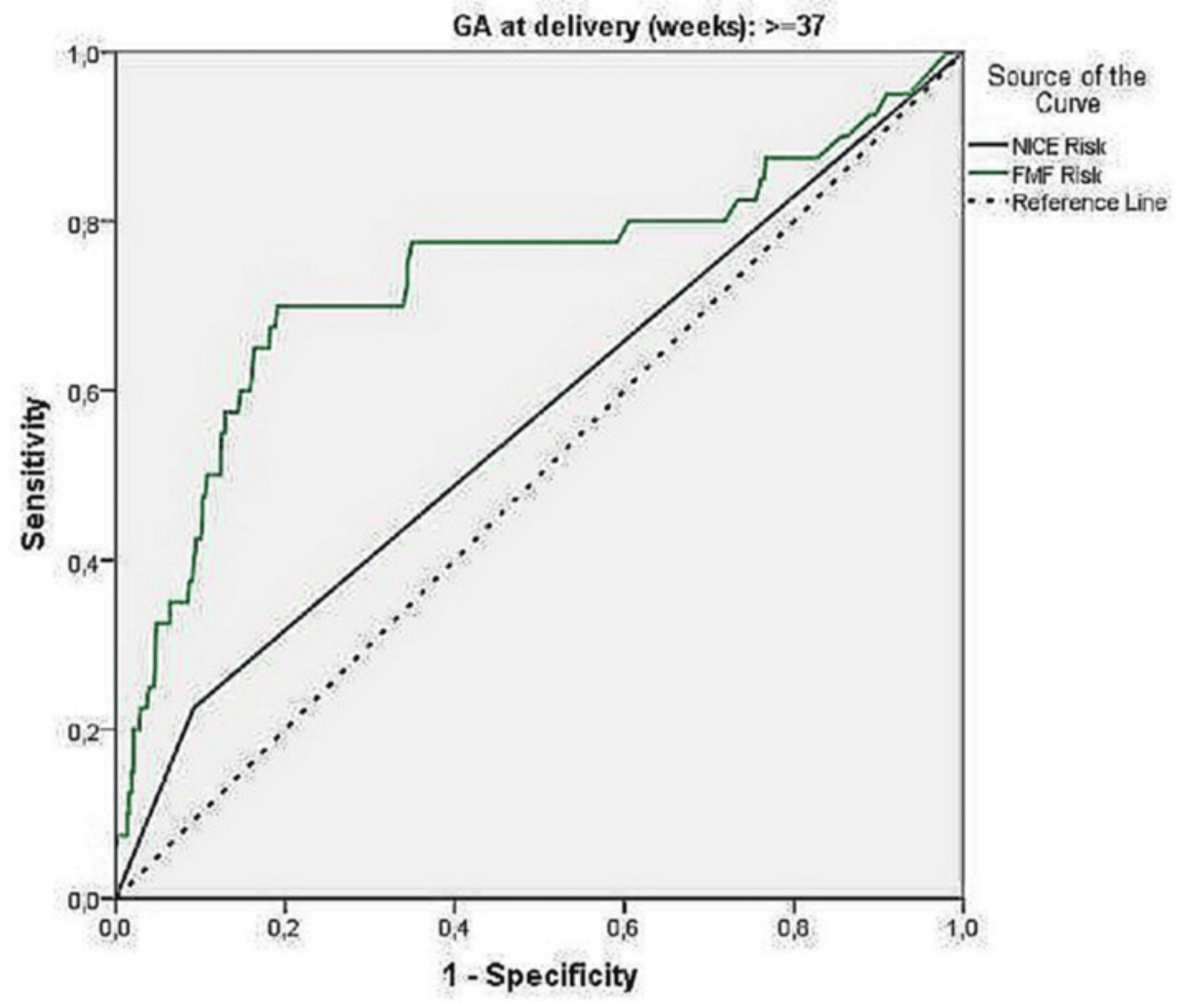
ROC curve for the predictive ability of NICE risk and FMF risk indices for preeclampsia ≥ 37 weeks (FMF risk 165.5) ROC: receiver-operating characteristic; NICE: National Institute for Health and Care Excellence; FMF: Fetal Medicine Foundation

The NICE risk score did not significantly predict preeclampsia in the total sample or by gestational week. Conversely, the FMF risk index showed significant predictive value. For the entire sample, the optimal point was 165.5, with 70.5% sensitivity and 80.9% specificity. For gestational ages under 37 weeks, the optimal point was 74, with 75% sensitivity and 100% specificity. For gestational ages of at least 37 weeks, the optimal point was 165.5, with 70% sensitivity and 80.9% specificity. A secondary analysis, aiming for 90% specificity, found the FMF method had 50% sensitivity at a 1 in 100 cutoff, with 89.9% specificity, similar to the NICE method. However, the FMF method's low positive predictive value (0.27%) indicates a high false positive rate. ROC curves are provided in Figures 1-3.

Preeclampsia rates and results from univariate logarithmic regression models using NICE and FMF risk indicators are detailed in Table 5. In the total sample, high-risk participants according to the NICE risk score were 2.93 times more likely to develop preeclampsia than low-risk participants. Those with an FMF risk score below 165.5 were 10.1 times more likely to have preeclampsia compared to those above 165.5. The FMF risk - 100 cutoff showed an 8.90 times higher likelihood of preeclampsia, though not as strong as the 165.5 cutoff. All methods showed significant associations (p < 0.05) with preeclampsia risk, with the FMF risk method, particularly the 165.5 cutoff, being the most effective. For gestational ages of at least 37 weeks, high-risk NICE participants were 2.86 times more likely to develop preeclampsia, and those with FMF risk <165.5 were 9.86 times more likely.

**Table 5:** Rates of preeclampsia and logarithmic regression FMF: Fetal Medicine Foundation; NICE: National Institute for Health and Care Excellence *relative ratio (95% Confidence Interval) ^#^due to null cells cannot be calculated.

			Preeclampsia				OR (95% CI)*	P
			No		Yes			
			N	%	N	%		
Sample total	NICE Risk	Low risk	547	94.1	34	5.9		
		High risk	55	84.6	10	15.4	2.93 (1.37 – 6.24)	0.005
	FMF risk	>165.5	487	97.4	13	2.6		
		<165.5	115	78.8	31	21.2	10.1 (5.1 – 19.9)	<0.001
	FMF risk	> 100	541	89.9	22	50		
		<100	61	10.1	22	50	8.87 (4.64 - 16.95)	0.01
<37 weeks	NICE Risk	Low risk	25	89.3	3	10.7		
		High risk	2	66.7	1	33.3	4, 17 (0, 29 – 60, 9)	0.297
	FMF risk	>74	27	87.1	4	12.9		
		<74	0	0.0	0	0.0	-^#^	-
>= 37 weeks	NICE Risk	Low risk	522	94.4	31	5,6		
		High risk	53	85.5	9	14.5	2.86 (1.29 – 6.33)	0.010
	FMF risk	>165.5	465	97.5	12	2.5		
		<165.5	110	79.7	28	20.3	9.86 (4.86 – 20.0)	<0.001

## Discussion

Preeclampsia affects 2-8% of pregnancies worldwide, causing significant maternal and perinatal complications. While aspirin administration between 11-14- and 36 weeks gestation can reduce premature preeclampsia risk in high-risk women, effective screening methods are crucial. Beyond its direct effects on pregnancy and associated maternal and fetal complications, preeclampsia imposes significant financial burdens on healthcare institutions. Overall, the prevention of preeclampsia is a critical priority for health systems.

With regards to the screening for preeclampsia in the first trimester, the NICE guidelines rely on maternal characteristics and medical history for risk assessment, whereas the FMF algorithm incorporates additional biochemical markers (PLGF or PAPP-A) and ultrasound parameters (UtA-PI). Both methods are applied in the 1st trimester, between 11-14 weeks of gestation, and in women considered high risk, aspirin is prescribed to prevent preeclampsia, especially early onset before 34 weeks of gestation.

Our study aimed to evaluate the preeclampsia screening efficacy of NICE guidelines versus the mini-combined test at 11-14 weeks. Exclusion criteria included missing data, advanced gestational age, fetal pathology, or twin pregnancy, and incomplete digital records. The study, as a Quality Improvement Project using a retrospective design, has several limitations that should be acknowledged. First, COVID-19 measures led to the omission of MAP measurements, which are a critical component of the mini-combined screening method. In addition, laboratory constraints prevented the inclusion of PLGF in the FMF algorithm, necessitating a simplified method comparison under these less-than-ideal conditions. Moreover, our study sample was predominantly drawn from a white population, which may limit the generalizability of our findings. Emerging research in more diverse populations has highlighted potential variations in preeclampsia screening efficacy, indicating that replication of this study in larger and more ethnically heterogeneous cohorts is essential to confirm and extend the applicability of our results.

Despite these limitations, our study benefits from being conducted in a real-world clinical setting, offering genuine insights into preeclampsia screening in routine practice. The quality improvement approach facilitated a rapid assessment of current methods and the identification of practical enhancements, especially valuable in resource-limited settings. Our findings contribute to evidence supporting a risk-based screening strategy, providing clinicians with practical guidance when ideal conditions are unattainable.

Our study included 646 pregnant women from May 4, 2021, to September 1, 2021, with births until March 18, 2022. We observed 44 cases of preeclampsia, 18 of gestational hypertension, 85 of gestational diabetes, and 6 of pregnancy cholestasis. 388 women had a BMI > 25, with 81 having a BMI > 35. There were 60 underweight newborns (12 with preeclampsia) and 23 NICU admissions. We recorded 31 preterm births (11 before 34 weeks, 20 between 34-36+6 weeks), one stillbirth, and one early intrauterine death. The population was generally low-risk: 93.2% white, average age of 30.4 years, and mostly without underlying diseases. 54% were multiparous, 0.8% had a family history of preeclampsia, and 96.9% conceived naturally. However, 60.1% were overweight or obese.

With regards to the prediction of preeclampsia, the NICE Risk Index showed no significant predictive value, with 22.7% sensitivity and 90.9% specificity. However, the FMF risk index demonstrated significant predictive value. For the entire sample, the optimal point was 165.5, yielding 70.5% sensitivity and 80.9% specificity. In pregnancies under 37 weeks, the optimal point was 74, with 75% sensitivity and 100% specificity. For pregnancies 37 weeks or later, the optimal point remained at 165.5, with 70% sensitivity and 80.9% specificity. The FMF method predicted all four preeclampsia cases before 37 weeks, while NICE predicted only one. A secondary analysis, aiming for 90% specificity, found the FMF method achieved 50% sensitivity at a 1 in 100 cutoff, with 89.9% specificity which is comparable to NICE.

In clinical practice, the choice between these methods would depend on the specific context: (a) If the priority is to identify as many potential cases of preeclampsia as possible (even at the cost of more false positives), the FMF risk method with a cutoff of 165.5 would be preferred. (b) If minimizing false positives is crucial, the FMF risk method with a cutoff of 100 might be considered, but its very low PPV is a significant drawback. (c) The NICE risk method, despite its high specificity, may not be reliable enough given its low sensitivity and lack of statistical significance.

The results of our study are in agreement with other international studies. More specifically, Guy et al., [17] conducted a prospective and larger study, with a sample of 7720 pregnant women, including in the screening method the maternal characteristics, MAP, PAPP-A, and UtA - PI, showed that for preeclampsia before 37 weeks the NICE method had 36.9% sensitivity with 84.1% specificity while the combined method with cutoff 1 in 100 had a sensitivity of 70.4% with a sensitivity of 81.9%. Regarding preeclampsia after 37 weeks, the sensitivity of the NICE method was 37.1% with a specificity of 79%, while the FMF method with an optimal point of 1 in 100, had a sensitivity of 49.% with a specificity of 82.2%.

Looking into the literature, the addition of PLGF to the combined method has a positive effect on its predictive value performance. Wright et al, [18] showed that the benefit of adding PLGF over PAPP - A is two-fold or even three-fold (almost a 5-15% increase) in predictive value in combinations with maternal characteristics or maternal characteristics plus MAP and/or plus UtA-PI.

The combined method as per the FMF algorithm, even in its simplified form, demonstrates superior predictive value compared to the NICE method. Our findings align with international studies, confirming the FMF algorithm's effectiveness in preeclampsia screening and potentially improving maternal and neonatal outcomes through more accurate risk assessment. Ideally, the comprehensive FMF model - including both MAP and PLGF measurements - should replace the NICE screening method for preeclampsia in 1st trimester, as endorsed by international literature and the FMF.

## Conclusions

This study demonstrates that the mini-combined method using the FMF algorithm substantially outperforms the NICE method, which showed low sensitivity and lacked statistical significance in screening effectiveness. Based on our results, aligning with the international literature, we strongly recommend adopting the complete combined method, including MAP and PLGF parameters, for screening for preeclampsia in the first trimester, which provides more accurate risk stratification. The integration of such advanced screening protocols into routine obstetric care would ultimately improve outcomes for mothers and infants while reducing the healthcare burden.
